# Human GMDS gene fragment hypermethylation in chronic high level of arsenic exposure with and without arsenic induced cancer

**DOI:** 10.1186/2193-1801-2-557

**Published:** 2013-10-24

**Authors:** Sarmishtha Chanda, Uma B Dasgupta, Debendranath Guha Mazumder, Jayita Saha, Bhaskar Gupta

**Affiliations:** Department of Biophysics, Molecular biology & Genetics, University of Calcutta, Kolkata, West Bengal 700092 India; Department of Physiology, Presidency University, Kolkata, West Bengal 700073 India; Department of Gastroenterology, Institute of Post-Graduate Medical Education & Research, Kolkata, West Bengal India; DNGM Research Foundation, Kolkata, West Bengal India; Department of Biotechnology, Presidency University, Kolkata, West Bengal 700073 India

**Keywords:** Arsenic exposure, Arsenic induced cancer, GMDS gene hypermethylation

## Abstract

Arsenic, though a poor mutagen, is an accepted environmental carcinogen. Perturbation of DNA methylation pattern leading to aberrant gene expression has been hypothesized as the mechanism for arsenic induced carcinogenesis. We had earlier demonstrated the hypermethylation of promoter region of p53 and p16 genes in persons exposed to different doses of arsenic. Till now no genomic hot spot has been identified which is frequently hypermethylated or hypomethylated in persons chronically exposed to environmental arsenic. In the present work, we have identified one hypermethylated sequence by methyl-sensitive arbitrarily primed polymerase chain reaction in the peripheral blood leukocyte DNA of chronically arsenic exposed persons with and without arsenic induced skin cancer. The sequence is from GMDS gene responsible for fucose metabolism. Southern hybridization of the sequence to the amplification products of methyl sensitive restriction enzyme digested genome of persons exposed to different doses of arsenic indicated that methylation increased in a dose dependent manner.

## Introduction

According to (International agency for research on cancer 1997) and National Research Council (NRC 1999) arsenic is an important environmental toxicant and carcinogen. However, the mechanism of arsenic mediated carcinogenesis is not clear as arsenic is a poor mutagen (Rossman et al. 1980; Jacobson and Moltanbano 1985; Lee et al. 1985) and does not induce significant point mutations. Biotransformation of arsenic, on the other hand, involves methylation of inorganic arsenic to organic monomethyl arsonic acid (MMA) and dimethyl arsinic acid (DMA), using the same methyl donor S-Adenosyl methionine (SAM) also involved in DNA methylation (Vahter 1999). The interference of the DNA methylation pathway with arsenic detoxification pathway, as both the pathways require SAM, can lead to aberrant DNA methylation, resulting aberrant expression and/or silencing of genes Goering et al. ( 1999). Therefore, epigenetic alterations, particularly aberrant DNA methylation has been mooted as a possible mechanism of arsenic induced carcinogenesis (Ren et al. 2010; Reichard and Puga 2010).

Cytosine-5 methylation at the CpG islands in the regulatory sequence of a gene is one of the key mechanisms of gene inactivation. DNA methylation/demethylation seems to regulate a plethora of biological processes involving transcription, differentiation, development, DNA repair, recombination, and chromosome organization. Perturbation of DNA methylation has been correlated with many cases of cancer (Jones and Baylin 2002). The hypothesis that arsenic perturbs DNA methylation has been tested successfully on tissue culture system (Mass and Wang 1997), and later we demonstrated hypermethylation of the promoter region of *p53* and *p16* genes in DNA extracted from peripheral blood leucocytes of persons exposed to different doses of arsenic (Chanda et al. 2006). A few highly exposed persons also showed *p53* hypomethylation (Chanda et al. 2006). Further arsenic induced genome wide hypermethylation has been demonstrated by us in DNA extracted from same population Majumder et al. ( 2010).

In this report we have further evaluated the hypothesis on a subsection of exposed population studied by isolating hypermethylated sequence from genomic DNA of arsenic exposed persons by methyl sensitive arbitrarily primed polymerase chain reaction (MS-AP-PCR). Differentially methylalated fragments have been identified and isolated from chronically arsenic exposed people. 7 persons of arsenic induced skin cancer out of 16 (all the cancer patients were recruited from previous study, Chanda et al. 2006), have one common hypermethylated fragment of 565 bp (this was cloned and sequenced). The same sequence was also isolated from 3 chronically arsenic exposed persons out of 10 (all of these 10 subjects were recruited from previous study, Chanda et al. 2006). The sequence is then analysed by bioinformatic tools (NCBI BLAST) to indicate that the fragment is actually situated in the human GDP mannose 4–6 dehydratase gene (*GMDS* gene). Southern hybridization of this fragment to amplified products from methyl sensitive restriction enzyme digested genomic DNA of persons exposed to arsenic in drinking water indicated that the sequence is indeed hypermethylated. The product of the identified gene is involved in fucose metabolism and it is reported that deletion of this gene results in cancer progression (Thompson et al. 1992; Becker and Lowe 2003;Yuan et al. 2008). Though have been found here in small proportion this hypermethylated fragment may be act as a potential target (probe) for detecting aberrant methylation in chronic high level of arsenic exposure.

## Materials and methods

### Subject selection

Subjects of this study were the same set of our earlier study on arsenic induced DNA hypermethylation in *p53* and *p16* gene promoter region and are all residents of South & North 24 Parganas, West Bengal, India (Chanda et al. 2006).

Criteria of diagnosis of arsenicosis and its severity are based on the parameters described earlier (GuhaMazumder et al. 1998; GuhaMazumder 2001; Chanda et al. 2006). In this study only the subjects for *p53* gene promoter hypermethylation group of the previous study had been chosen. Participants had been divided into the five groups A, B, C, D according to the concentration of arsenic in their drinking water, i.e. 0–50, 51–250, 251–500, 501–1000 μg/l respectively as earlier and group E with 500–1100 μg/l of arsenic suffering from arsenic induced skin cancer. As the concentration of arsenic in group A is within the permissible limit according to WHO and Medical Council of India, it was considered as the unexposed control group (NRC 1999). Initially the number of participants in each group was 24, 12, 18, 15 and 16 in A, B, C, D and E group respectively (Chanda et al. 2006). Among those, 11 subjects in group C, 10 subjects in group D and all the 16 subjects in group E were chosen for this study. All of the subjects chosen for MS-AP PCR have hypermethylated *p53* promoter region compared to normal unexposed persons (Chanda et al. 2006). All of the subjects studied for MS-AP-PCR were compared to normal unexposed subjects treated in similar way. In this study 12 of the normal unexposed subjects were recruited from group A.

The initial studies on isolation of hyper/hypo methylated stretch of genomic DNA was performed with peripheral blood leukocyte DNA of highly arsenic exposed persons of group D and arsenic induced cancer group, E. Later, lower exposure group C was also evaluated for the presence of such hyper/hypomethylated gene fragments. Among 16 of the cancer patients (group E) studied, 7 had a hypermethylated DNA fragment of 565 nucleotide long sequence. Among 10 subjects of group D, 3 had that hypermethylated fragment. The fragment identified was from the participants of group D and group E but not from any lower exposure group. Although there is an overlap between group D and E in respect to the concentration of arsenic in water but the difference is one group have arsenic induced skin cancer with higher degree of skin manifestations (group E) while the other group (group D) is only characterized by higher degree of skin manifestations without cancer. The identified and isolated hypermethylated fragment was then sequenced. The sequence is from the intronic region of human *GMDS* gene situated in between exon 1 and 2. The southern hybridization studies of the identified fragment with DNA of 4 persons, taken 1 from each exposure group indicate that the sequence is indeed hypermethylated. Demographic data for this study population is described in detail (Table 1). Once the fragment was identified and isolated from peripheral blood leukocyte DNA of arsenic induced cancer patients, the procedure was cross-checked using DNA samples isolated from cancer biopsy samples of the same patients. But it was not done in case of group D samples due to lack of biopsy tissues in those cases (as these are not arsenic induced cancer).

**Table 1:** **Demographic data of study subjects taken from different arsenic exposure groups**

Age group	Sex	Group A (0-50 μg/l) p53 methylation	Group C (250-500 μg/l) p53 methylation	Group D (501-1000 μg/l) p53 methylation	Group E (500-1100 μg/l) p53 methylation
<20 years	Male	N = 2; 0.08, 0.19	N = 2; 1.34, 1.62		
	Female		N = 2; 1.50, 1.45		
21-40 years	Male	N = 3; 0.03, 0.23, 0.15	N = 3; 1.50, 2.78, 5.00	N = 4; 1.95, 2.04, 4.3 2.46	N = 3; 0.85, 1.09, 1.32
	Female	N = 1; 0.15		N = 1; 2.56	N = 3; 1.00, 1.62, 1.09
41-60 years	Male	N = 3;0.18, 0.27, 0.27	N = 2; 1.56, 2.4	N = 2; 2.85, 4.46	N = 4; 1.42, 3.08, 2.09, 2.09
	Female	N = 3; 0.08, 0.09, 0.32	N = 2; 1.95, 4.66	N = 2 4.45, 2.7	N = 1; 2.55
>60 years	Male			N = 1; 2.15	N = 4; 2.20, 2.23, 1.62, 1.60
	Female				N = 1; 2.11
Smoking status	Smoker	8	9	7	11
	Nonsmoker	4	2	3	5
	Exsmoker				
Avarage duration of exposure		11.5 years	15 years	10 years	17 years
Total number of samples	47/M	12	11	10	16

Note: numerical values in each cell indicate the degree of p53 methylation for individual study subjects recruited in the study.

Written informed consent was obtained from all participants before drawing their blood. The name of the institute where human clinical studies were carried out is Institute of Post Graduate Medical Education and Research, Kolkata (IPGME&R), which is run by Govt. of West Bengal, a state government within the framework of Republic of India.

Molecular Biological and *in silico* experiments were carried out in University of Calcutta and Presidency University, Kolkata which are also run by Govt. of West Bengal. Ethical principles followed by the institute are guided by rules as formulated by Indian Council of Medical Research and these are in agreement with Helsinki declaration.

### Determination of Arsenic concentration in urine and water

Level of arsenic in drinking water and urine was determined by atomic absorption spectrophotometer with hydride generation system (AAS) Atallah and Kalman ( 1991).

### DNA isolation from blood

DNA was extracted from whole blood by conventional chloroform extraction method using 0.01% SDS and Proteinase K (0.1 mg/ml) (Miller et al. 1998).

### DNA isolation from tissue

DNA was extracted from cancer biopsy tissue samples by conventional phenol-chloroform (1: 1, v/v) extraction method and then by chloroform extraction followed by salting out using 0.01% SDS and Proteinase K (0.1 mg/ml) (Miller et al. 1998).

### *p53* methylation status analysis

The p53 tumor suppressor gene methylation status was analyzed in each subject by the method described earlier (Chanda et al. 2006).

### Determination of clinical symptom score

Each subject was assigned a clinical symptom score which reflects the severity of his/her skin manifestations. Both pigmentation and keratosis were graded as 1,2 or 3, depending on the level and severity of symptoms. Sum of the two was clinical symptom score, so that a person can have maximum score of 6. Control subjects have no pigmentation and keratosis and therefore have a clinical symptom score 0. The detail structure of the scoring system for pigmentation and keratosis is given in Table 2.

**Table 2:** **Dermatological criteria and graduation of chronic arsenic toxicity for scoring sustem of skin manifestations**

**Pigmentation**		
**Mild 1**	**Moderate score = 2**	**Severe score = 3**
Defuse Melanosis, Mild Spotty pigmentation, Leucomelanosis	Moderate Spotty pigmentation	Blotchy Pigmentation, Pigmentation of under surface of tongue, buccal mucosa
**Keratosis**		
**Mild Score = 1**	**Moderate score = 2**	**Severe score = 3**
Slight thickening, or minute papules (<2 cm) in palm and soles	Multiple raised keratosis papules (2 to 5 cm) in palm & soles with diffuse thickening	Diffuse severe thickening, large discreet or confluent keratotic elevations (>5 cm), palm and soles (also dorsum of extremely and trunk)

The underlined data represents the clinical symptom score.

### Restriction enzyme digestion for arbitrarily primed PCR

Concentration and quality of isolated genomic DNA was determined UV–vis spectrophotometer (OD 260/280 >1.8). 300 ng of total genomic DNA isolated from persons, unexposed/exposed to arsenic through drinking water, was digested with 5 units of RsaI and 5units of HpaII restriction enzyme at 37°C overnight. HpaII is a methylation sensitive isoshizomer of MspI whose recognition sequence is CCGG. A sequence hypermethylated at this site would not be digested, whereas the unmethylated DNA would. The persons taken for MS-AP-PCR were from higher exposure groups of arsenic (251–500 μg/l, i.e. group C; 500–1000 μg/l, i.e. group D; and arsenic induced cancer group, E, with an exposure level of 500–1100 μg/l) and all have hypermethylated *p53* promoter. Out of 18 in group C, 11 subjects were taken for MS-AP-PCR. All have hypermethylated *p53* promoter region. In group D only 10 samples were chosen with hypermethylated *p53* promoter having a median value of 2.63. In group E all the 16 samples were studied with *p53* promoter hypermethylation with a median value of 1.62. The median value for *p53* methylation in group A (unexposed control group) was 0.26 which is treated here as a basal value for normal unexposed persons (Chanda et al. 2006). Demographic data and *p53* methylation values for subjects included in this study are presented in Table 1.

### Methyl sensitive arbitrarily primed PCR (MS-AP- PCR)

When RsaI + HpaII digested DNA was used as template in MS-AP-PCR using random primers that target CG-rich DNA sequences (Zhong and Mass 2001), a series of amplified products were observed. Of these, a band present in PCR products of arsenic exposed DNA but absent in PCR products of similarly digested unexposed DNA represents the region of hypermethylation (Zhong and Mass 2001). We used 3 different primers. Amongst these primers, OPN Hind12 (5’-AGCTTCTCCCTC-3’) gave one common hypermethylated fragment in arsenic induced cancer patients and in highly arsenic exposed persons when subjected to PCR amplification. The concentration of OPN Hind 12 in the PCR reaction mixture was 0.5μM. The PCR protocol was initial denaturation at 94°C for 5 minutes, 35 cycles at 94°C for 1 min, 40°C for 1 min, 72°C for 2 min, followed by 10 min at 72°C.

### Isolation of candidate bands

The PCR products from DNA of arsenic exposed persons were compared with PCR products from control DNA. The band, which appears only in the exposed DNA but not in unexposed DNA was supposed to be region of hypermethylation. Similarly, band which appears only in the unexposed control but not in the exposed DNA indicated the site of hypomethylation in the DNA of exposed persons. Such a region of hypermethylation from chronically high arsenic exposed people with and without arsenic induced cancer was identified. The ethidium bromide stained PCR amplified DNA band was then excised from the gel by a scalpel and recovered by the usual 'crush and soak’ method (Sambrook et al. 1989). The candidate band isolated from subjects were from group D and E. Clinical symptom score, *p53* methylation status and degree of arsenic exposure for those subjects are given in Table 3.

**Table 3:** **Demographic data and p53 methylation status of subjects having GMDS gene hypermethylation**

Sample	Age (yr)/sex	Smoking status	Conc. of arsenic in water μg/l	Duration of exposure yrs	Degree of pigmentation	Total urinary arsenic μg/l	p53 methylation value
KA 261	52/M	Non smoker	580	7	++ + 3	272.8	4.46
Gr. D							
DHW 088	43/M	smoker	683	5	++++ 4	89	2.85
Gr. D							
CW045	38/M	smoker	531	5	++++ 4	189	2.46
Gr.D							
CNBB 33	48/F	Non-smoker	826	14	++++ 4	212	2.55
Gr.E							
CNBB 28	40/M	Ex smoker	740	10	++++ 4	126	1.62
Gr.E							
A 10	51/M	smoker	514	17	++++ ++ 6	211	3.08
Gr.E							
A 15	47/M	smoker	623	13	++++ ++ 6	97	2.09
Gr.E							
A 20	53/M	smoker	744	10	++++ 4	143	2.09
Gr.E							
A 17	63/M	smoker	556	17	++++ ++ 6	171	2.20
Gr.E							
A 21	61/M	smoker	631	10	++++ ++ 6	206	2.23
Gr.E							

Note: The pigmentation and keratosis was assigned as a numerical score according to the degree of severity.

### DNA cloning in plasmid vector

The gel- recovered PCR product was re-amplified using the same PCR protocol with same primer. The amplified product was then purified by ethanol precipitation and cloned in E.coli *XL1 blue* strain using *pTZ57R/T* vector (TA cloning kit, Fermentas). The positive clones were identified by performing colony PCR with universal primers and sequenced.

### Southern hybridization

Exactly equal amount (1.3 μg) of genomic DNA of four persons from four different exposure groups (Group A, B, C, D) were subjected to restriction digestion by RsaI and HpaII and incubated overnight at 37°C. Each of the digested products was then subjected to PCR amplification using primer OPN Hind12. The PCR products obtained from four different DNA samples were resolved by electrophoresis on a 2% agarose gel. The gel was blotted on nylon membrane using standard technique and then hybridized with α- P^32^ dCTP (BARC, India) labelled clone insert. The same procedure of hybridization was carried out using one DNA sample from cancer patient where instead of group A, B, C, D group B,C, D and E were used to hybridise with the labelled clone of the fragment isolated. The relevant parameters for the persons taken from four different groups for hybridization are described in Table 4.

**Table 4:** **Demographic data of subjects taken from different exposure group for southern hybridization**

Sample	Age (yr)/sex	Smoking status	Conc. of arsenic in water (μg/l)	Duration of exposure yrs	Degree of pigmentation	Total urinary arsenic (μg/l)	p53 methylation value
1	40/M	smoker	11	5	- (0)	0.0	0.20
2	47/M	smoker	118	5	+ (1)	31	0.78
3	52/M	smoker	314	7	+++ (4)	87	1.95
4	39/M	smoker	644	6	++++ (6)	223	2.56

## Result

Using the technique of MS-AP PCR, 1 common hypermethylated DNA fragment was identified from 10 different people with chronic high level of arsenic exposure with and with out cancer. Among 16 of the arsenic induced cancer patients studied (belonging to group E) 7 have the hypermethylated DNA fragment of 565 nucleotide base pair. Among 10 of group D subjects 3 have been identified to harbour this hypermethylated DNA fragment. Demographic data and *p53* methylation status for these 10 subjects (with hypermethylated DNA) has been listed in Table 3. Interestingly, people from lower arsenic exposure (group C) did not have this hypermethylated gene fragment.

The fragment identified is a region of hypermethylation in comparison to normal unexposed persons. DNA sequence analysis revealed that the identified fragment has significant homology match (99%) to the sequence of human *GMDS gene (*Accession no. NT_007592.15), *Homo sapiens*) (taken from GENEBANK database) after BLAST search. The sequence is situated in the intron between exon 1 and 2 of *GMDS* gene. (Genomic context: chromosome: 6; Maps: 6p24.1-25.3). It is the longest intronic sequence in *GMDS* gene (> 1,80,000 bp). This gene is involved in carbohydrate metabolism and generation of fucose. Fucose mediates initial contact between extravagating leucocytes and endothelial cells. Influence of fucose generating enzymes on leukocyte adhesion activity has been reported (Sullivan et al. 1998; Eshel et al. 2001).

**Figure 1:**
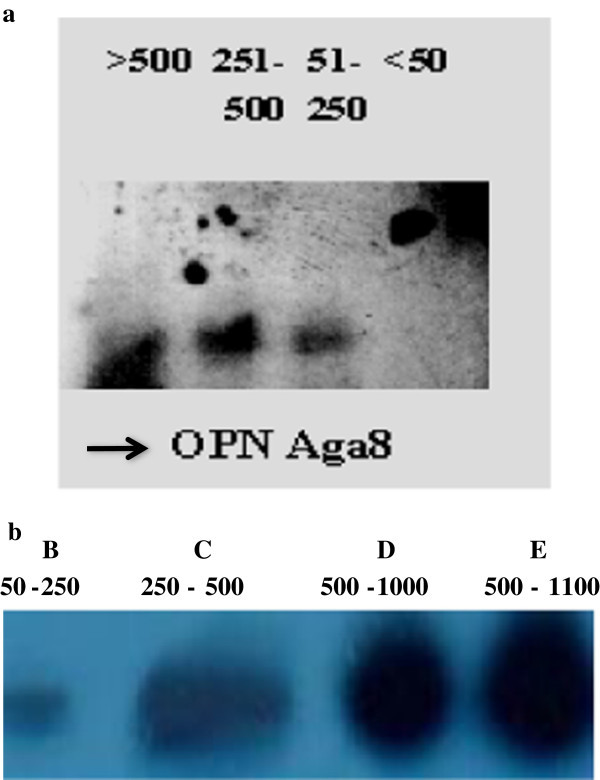
**Represents the southern blots of cloned insert. a.** Southern hybridization pattern of the cloned insert (OPN Aga8) to amplification products of HpaII digested DNA from four persons of different exposure groups. **b.** Southern hybridization pattern of the cloned insert (OPN Aga 8) to amplification products of HpaII digested DNA from four persons of three different exposure groups without arsenic induced cancer and one group of arsenic induced cancer.

When the insert of the clone is hybridized to the PCR products amplified by the OPN Hind 12 primer from HpaII digested genomic DNA of persons exposed to various doses of arsenic and arsenic induced cancer, it was found that the hybridization increases in higher exposure groups and in arsenic induced cancer patients. This indicates that the segment is indeed hypermethylated in genomic DNA of persons with high arsenic exposure and also in arsenic induced cancer patients (Figure 1a, 1b). Hypermethylation rendered the fragment insensitive to digestion by the methyl sensitive enzyme HpaII at the relevant site in higher exposure group DNA and the desired region was available for amplification. So the amount of PCR product template available for associating with the probe is more in the arsenic exposed group and in cancer group with hypermethylation in their p53 promoter region than in the unexposed group.

Our identified fragment, OPN Aga 8, shows very low association with the DNA of <50 μg/l exposure group (Figure 1a). The degree of association increases gradually with the degree of arsenic exposure in higher exposure groups. The fragment isolated has a higher degree of association during hybridization with PCR product of person with arsenic induced cancer (Figure 1b).

**Figure 2:**
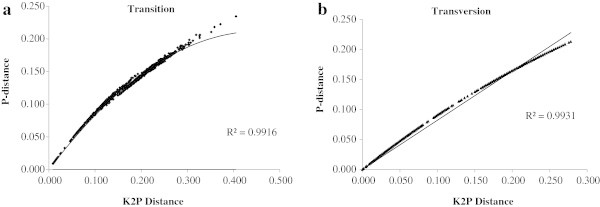
**Pairwise sequence divergence among the 42 animal taxa.** GMDS mDNA, **(a)** and **(b)**; plots of Kimura 2 parameter (K2P) inferred Transition (Ts) and Transversion (Tv) distances against the *P-*distance.

**Figure 3:**
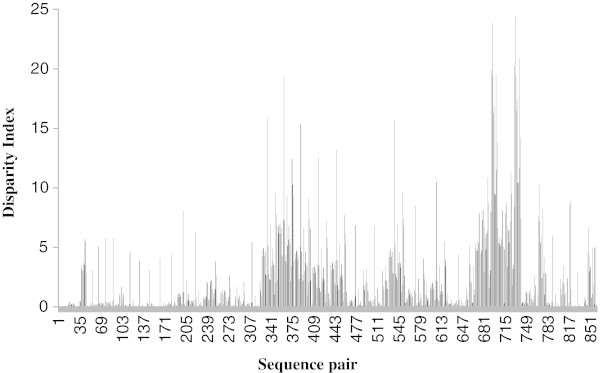
**Disparity Index per site is shown for all sequence pairs for GMDS sequences.** All positions containing gaps and missing data were eliminated. Values greater than 0 indicate the larger differences in base composition biases than expected based on evolutionary divergence between sequences and by chance alone. The analysis involved 42 nucleotide sequences. There were a total of 670 positions in the final dataset.

Our results indicated that in the *GMDS* mRNA dataset, non-linearity was found when pair wise sequence divergence per site corrected from Kimura-2-parameter (K2P) distances were plotted against p-distances for both transition and transversion among the studied animal species, but Ts sites exhibited slightly higher saturation over Tv (Figure 2a and 2b). We have estimated Disparity Index (DI) per site of *GMDS* mRNA sequences between all sequence pairs Kumar and Gadagkar ( 2001). Value greater than '0’ indicate larger difference in base composition biases, than expected. This is based on evolutionary divergence between sequences and by chance alone. There were a total of 670 positions in the final dataset. Highest Disparity Index was observed between *Apis florae* and *Anopheles gambiae* (DI = 24.38) among the other sequence pairs (Figure 3).

### Phylogenetic analysis

Human *GMDS* gene sequence comparison was done with 42 different species of different animal groups, for which the sequences available in GenBank gives us the evolutionary relationship of this gene among the selected species (Table 5). Multiple sequence alignment was executed with three dataset derived from *GMDS* mRNA sequences using Clustal W and it was found that the sequence identified is conserved in a number of genera studied. The sequence was further analyzed using the Kimura 2-parameter model, p-distance to assay the probability of the number of transitional and transversional substitutions per site between sequences. All positions containing gaps and missing data were eliminated. Phylogenetic tree was constructed based on Neighbor-Joining method (NJ) with Kimura 2-parameter using MEGA version 5.05 Tamura et al. ( 2011; Saha et al. 2013a, b) from both transition and transversion data. Standard error estimate(s) were obtained by bootstrap procedure (1000 replicates). Disparity Index per site was estimated for all sequence pairs Kumar and Gadagkar ( 2001).

**Table 5:** **GenBank accession numbers and size of GMD sequence of sampled taxa**

Sr. No.	Organism	Accession No.	Size
1	*Homo sapiens*	BC000117.1	1119 bp
2	*Pan troglodytes*	XM_518203.3	795 bp
3	*Pongo abelii*	XM_002816345.1	1119 bp
4	*Nomascus leucogenys*	XM_003272184.1	1119 bp
5	*Macaca mulatta*	NM_001266789.1	1119 bp
6	*Callithrix jacchus*	XM_002746279.2	1119 bp
7	*Equus caballus*	XM_001490703.3	1050 bp
8	*Ailuropoda melanoleuca*	XM_002922834.1	1119 bp
9	*Canis lupus*	XM_545311.3	1011 bp
10	*Loxodonta africana*	XM_003417823.1	1119 bp
11	*Cavia porcellus*	XM_003463230.1	1050 bp
12	*Bos taurus*	NM_001080331.1	1119 bp
13	*Oryctolagus cuniculus*	XM_002720970.1	1662 bp
14	*Cricetulus griseus*	NM_001246696.1	1119 bp
15	*Rattus norvegicus*	NM_001039606.1	1119 bp
16	*Mus musculus*	BC093502.1	1119 bp
17	*Ornithorhynchus anatinus*	XM_001510089.1	1260 bp
18	*Anolis carolinensis*	XM_003225586.1	1006 bp
19	*Taeniopygia guttata*	XM_002197547.1	1035 bp
20	*Gallus gallus*	XM_418977.3	1086 bp
21	*Meleagris gallopavo*	XM_003204780.1	1047 bp
22	*Xenopus laevis*	BC157411.1	1110 bp
23	*Danio rerio*	NM_001102475.2	1113 bp
24	*Salmo salar*	NM_001141373.1	1113 bp
25	*Oreochromis niloticus*	XM_003457295.1	1116 bp
26	*Nematostella vectensis*	XM_001622499.1	1077 bp
27	*Trichoplax adhaerens*	XM_002116109.1	1080 bp
28	*Brachionus manjavacas*	FJ829249.1	1027 bp
29	*Culex quinquefasciatus*	XM_001868832.1	1107 bp
30	*Anopheles gambiae*	XM_308963.3	1089 bp
31	*Aedes aegypti*	XM_001650058.1	1149 bp
32	*Tribolium castaneum*	XM_968229.1	1071 bp
33	*Drosophila willistoni*	XM_002066598.1	1194 bp
34	*Amphimedon queenslandica*	XM_003384374.1	1110 bp
35	*Brugia malayi*	XM_001898680.1	1164 bp
36	*Loa loa*	XM_003138092.1	1143 bp
37	*Dictyostelium purpureum*	XM_003283436.1	1068 bp
38	*Acyrthosiphon pisum*	XM_001949034.2	1086 bp
39	*Nasonia vitripennis*	XM_001605356.2	1071 bp
40	*Megachile rotundata*	XM_003702009.1	1077 bp
41	*Bombus impatiens*	XM_003484683.1	1071 bp
42	*Apis florea*	XM_003692995.1	1077 bp

Maximum Composite Likelihood Estimate of the pattern of nucleotide substitution was estimated according to Tamura et al. ( 2004) where each entry shows the probability of substitution (r) from one base (row) to another base (column) (Table 6). For simplicity, the sum of r values is made equal to 100. Rates of different transitional substitutions are shown in **bold** and those of transversional substitutions are shown in *italics*. The nucleotide frequencies are 28.32% (A), 26.46% (T/U), 24.03% (C), and 21.20% (G). The transition/transversion rate ratios are *k1* = 1.887 (purines) and *k2* = 3.132 (pyrimidines). The overall transition/transversion bias is *R* = 1.219, where *R* = [A*G*k1 + T*C*k2]/[(A + G)*(T + C)]. The whole analysis involved 42 different nucleotide sequences. All positions containing gaps and missing data were eliminated. There were a total of 670 positions in the final dataset.

**Table 6:** **Maximum composite likelihood estimate of the pattern of nucleotide substitution**

	A	T	C	G
**A**	**-**	*5.91*	*4.73*	**10.12**
**T**	*6.32*	**-**	**14.82**	*5.36*
**C**	*6.32*	**18.5**	**-**	*5.36*
**G**	**11.93**	*5.91*	*4.73*	**-**

Note: Specificity for the bold symbols are justified in result.

**Figure 4:**
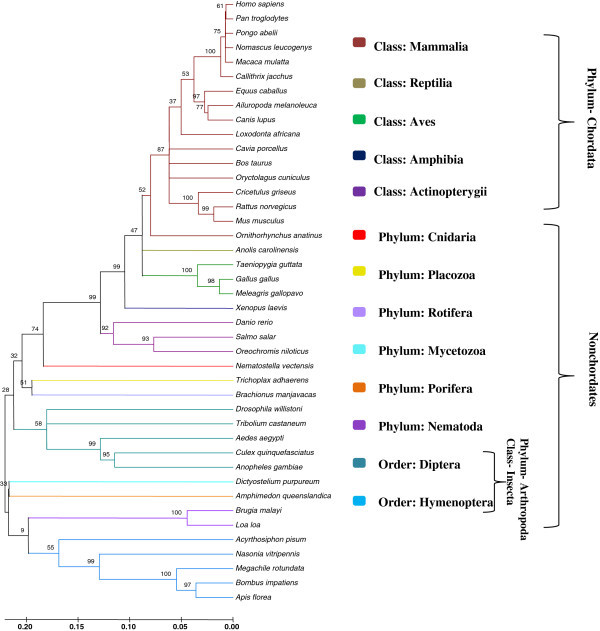
**Unrooted neighbor-joining tree constructed from the GMDS mDNA sequences (branch length = 4.18962548).** The percentage of replicate trees in which the associated taxa clustered together in the bootstrap test (1000 replicates) is shown above the branches. The evolutionary distances were computed using the Kimura 2-parameter (K2P) method and are in the units of the number of base differences per site. There were a total of 670 positions in the final dataset.

*GMDS* mRNA derived phylogenetic tree inferred by the NJ method represented in Figure 4. Due to saturation of both the substitutions, the sum of the transition and transversion for phylogenetic tree reconstruction by the NJ method based on K2P model has been used. These sequences are composed of 435 variable sites and 389 parsimony informative sites. The transition/transversion ratio and the overall mean distance has been found to be 0.96 and 0.333 ± 0.016. We have observed that *GMDS* sequence of *Callithrix jacchus* shared a common ancestor with the sister clade containing *Homo sapiens, Pan troglodytes, Pongo abelii, Nomascus leucogenys* and *Macaca mulatta* with 100% bootstrap support. *Equus caballus* have shown monophyly with closely related sister species *Ailuropoda melanoleuca* and *Canis lupus* which was supported by high bootstrap value (97%). *Cricetulus griseus, Rattus norvegicus* and *Mus musculus* formed a monophyletic group with high bootstrap support (100% and 99% respectively), whereas *Ornithorhynchus anatinus* diverged early in the tree among all other mammamls under study. NJ tree also depicted that *Taeniopygia guttata, Gallus gallus* and *Meleagris gallopavo* belong to the class Aves that have exhibited monophyletic origin and evolved parallely with the reptiles (*Anolis carolinensis*) but diverged after the class Amphibia and Actinopterygii. Class Insecta belongs to the phylum Arthropoda consisted of two clades, one of order Diptera and the other of Hymenoptera. Two Nematod species, *Brugia malayi* and *Loa loa* are closely related providing 90% sequence similarity.

### Identified sequence OPN Aga 8

TCCCTCACTA CTCAAAGTTG ATGACTTCTT AAACCAAAAT GGTTGGTCAG AATCCAATCA AGAATATAAA GGCAACTGAA TAAATAAAAC CATAAAGTAA GTGTAAAATA CTAGTGTCCA GACATCTGAG ATGTATGTGG CTACTATGAA ACTTCCACAG CTGTACCGGC CGGGAGCTCA CGTGGTTCCC CAGGTTTAAC AGAACCCATT ACCAGTAAGA GTTTTATTTG CTTAATAAAT TACATTCTAA AGCACAATAG CCTAGGCTCA TAGCTGTAAA ATTGCCAAAT ATTGTCAATG ACCACTCTCT GGTCATAAAT AACAAAATAA TCTTGTGACT CATTGGATTT TTGATTCCCAAGGCGATTCT TTCTCGCCAT TACTCAAAAA TGTGAAAAAG TGCCTCTACGTGGCATTTTA TGGAGGATAT AAATTACTCA AAGGAGATGA CATAGGACAGATTTGTAGGC CGAGTAACAG GAACCAGCCA ACCAACTGTG TAAATTAAAGAACTAGTGAC AAAGAAGAGG GCTAGTGAAA GAATTCTGAA ATCCTAAGAA CAGAT

## Discussion

The mechanism by which arsenic contributes to the development of cancer is currently a subject of intense interest. Arsenic does not act as a point mutagen. However, metabolism of arsenic involves methylation of inorganic arsenate to dimethyl arsinic acid via alternating reduction of pentavalent arsenic to trivalent arsenic and addition of methyl group (Vahter 1999; Donohue and Abernathy 2001). The arsenic methyl transferase uses the same methyl donor SAM as DNA methyltransferase (Dnmt) and other methyltransferases. Interaction of arsenic methylation/detoxification pathway with DNA methylation pathway and consequent imbalance in DNA methylation has been envisaged. Increase of cytosine methyltransferase transcript after arsenic exposure has been reported (Zhong et al. 2001), and this might explain the initial hypermethylation through excessive induction of the enzyme. On the other hand prolonged arsenic exposure may cause depletion of the SAM pool due to over consumption of the methyl groups by arsenic methyltransferase, and cause hypomethylation of DNA. Although we have failed to isolate any hypomethylated fragment from patients who have arsenic induced cancer or persons having chronic high level of exposure with systemic manifestations, yet, there was number of subjects with *p53* promoter hypomethylation in our previous study (Chanda et al. 2006). In fact decrease of tissue arsenic burden has been correlated with methionine intake in experiments with laboratory rats exposed to arsenic (Nandi et al. 2005). Interestingly the fragment of *GMDS* gene isolated from subjects having high degree of arsenic exposure and relatively high degree of *p53* methylation are from male subjects except one. We had not found any correlation between sex and *p53* methylation status in our previous study (Chanda et al. 2006). The hypermethylated gene fragment has been isolated from both smokers and nonsmokers although in the present study the number of smoker having GMDS gene intron hypermethylation is 8 out of 10. In our previous study we showed that there was no association between the p53 and p16 methylation status with individual’s smoking habit. In this study, due to small sample number, we could not carry out statistical evaluation for correlation between smoking habit and GMDS gene methylation. The fragment isolated in this study is from 9 male subjects and only one female subject out of 10 subjects. Increase in number of study subjects and consequent rise in the number of subjects having *GMDS* gene hypermethylation may provide definite information about the association (if any) of sex specificity of *GMDS* gene hypermethylation with arsenic exposure in human.

Such aberrant methylation of the genome leading to gene expression anomalies and have been mooted as possible mechanism of arsenic induced carcinogenesis. Cancer, which results from inappropriate expression of genes, may involve hypomethylation of protooncogene and/or hypermethylation of tumor suppressor genes that can alter the level of their expression and thereby promote cancer. In fact, there are recent observations that widespread methylation changes occur during tumor development (Jones and Baylin 2002).

Overall, tumor cell DNA is hypomethylated compared to normal cell DNA and underexpression of *Dnmt1* gene causes aggressive tumor induction in genetically engineered mice (Gaudet et al. 2003). However, for some tumor suppressors like *p16, p15* methylation is a common alternative to point mutation and in others like *RASSF1A* or *H1C1*, it is the only mechanism for tumor specific loss of function (Jones and Baylin 2002). Silencing of genes like *TIMP-3* through methylation has been associated with metastasis (Darnton et al. 2005).

Methylation of DNA is maintained by a balance of the activity of DNA methyltransferase (Dnmt 1, Dnmt 3a and Dnmt 3b) and DNA demethylase (mbd2) activity. Inhibition of mbd2 by antisense expression results in inhibition of anchorage-independent growth of antisense transfected cancer cells or cells infected with an adenoviral vector expressing antisense *mbd2* (Slack et al. 2002). Expression of *Dnmt* mRNA is significantly high in gastric cancer in comparison to non-cancerous gastric mucosa (Fang et al. 2004). Similarly level of *mbd2* mRNA level is significantly lower in gastric cancer tissue than normal gastric mucosa (Fang et al. 2004).

Previous works with human adenocarcinoma cell line in tissue culture showed that arsenic induces significant changes in methylation status in tumor suppressor gene *p53* Mass and Wang ( 1997). Later, using arsenic exposed human kidney cell lines global hyper and hypomethylation has been demonstrated by the same group (Zhong et al. 2001). DNA sequencing and SssI methylase assay were used for estimation of genomic CpG methylation level. Arsenic exposure of A 549 cells in culture resulted in a dose dependent increase in cytosine methylation in *p53* gene and a small increase in global methylation Mass and Wang ( 1997). Later we have shown that arsenic induces genomic hypermethylation in chronically exposed persons Majumder et al. ( 2010). An increase in the rate of transcription of DNA methyltransferase gene in cells exposed to arsenite was detected by RT-PCR (Zhong et al. 2001). Our group has demonstrated for the first time that there is dose dependent enhancement of methylation in the promoter region of *p53* and *p16* tumor suppressor genes of genomic DNA extracted from peripheral blood leucocytes of persons exposed to various doses of arsenic (Chanda et al. 2006). However, both these genes are associated with cell cycling and repair, and the possibility exists that methylation perturbation observed is engineered through disturbances in cell cycle produced by arsenic, and is local, rather than a global effect of arsenic.

In the present work we have investigated that whether there is any probable common target for aberrant DNA methylation after arsenic exposure in exposed persons apart from *p53* or *p16* gene methylation. We have successfully identified one fragment of hypermethylated DNA from persons exposed chronically to arsenic and persons having arsenic cancer. The subjects have been chosen from our previous study population (Chanda et al. 2006) having hypermethylated p53 promoter region with chronic high level of arsenic exposure with and without arsenic induced cancer. Therefore persons having GMDS gene intron hypermethylation also have p53 promoter hypermethylation. Thus this study reflects an association between the p53 promoter hypermethylation with GMDS gene intron hypermethylation in chronic high level of arsenic exposed people. The fragment was isolated from both peripheral blood leukocyte DNA and from cancer biopsy tissue of persons having arsenic induced cancer. The hypermethylated DNA fragment is from *GMDS* gene responsible for fucose metabolism. GMDS is the binding partner of tankyrase which is needed to be associated for the first step of fucose biosysnthesis (Bisht et al. 2012). Oligosaccharides are involved in various aspects of life process including birth, differentiation, growth, inflammation, carcinogenesis, and cancer metastasis. Fucosylation is one of the most important oligosaccharide modifications in cancer. This type of glycomodification can be treated as a biomarker in cancer (Moriwaki et al. 2009; Miyoshi et al. 2012).

Fucosylated alpha-fetoprotein (AFP) is widely used in the diagnosis of hepatocellular haptoglobin have also been found in sera of patients with various carcinomas (Miyoshi et al. 2012). Deletion mutation of the *GMDS* gene plays a pivotal role in fucosylation in human colon cancer. Loss of function mutation of this gene may lead to a virtually complete deficiency of cellular fucosylation, tumor progression and metastasis (Nakayama et al. 2013) and transfection of the wild-type *GMDS* into *HCT116* cells restored the cellular fucosylation. This type of *GMDS* mutation resulted in resistance to *TRAIL*-induced apoptosis followed by escape from immune surveillance (Moriwaki et al. 2009; Haltiwanger 2009; Moriwaki et al. 2011) and thus promote carcinogenesis. Further, epigenetic regulation of fucosylation and TRAIL induced apoptosis in conjunction to cancer had been studied by same group (Moriwaki et al. 2010). Although in the present study we have not shown any association with the level of fucose in patients with *GMDS* gene hypermethylation, but still *GMDS* gene fragment hypermethylation is associated with *p53* hypermethylation with development of arsenic induced cancer (in group E) or severe skin manifestations (in group D) as a result of chronic high level of exposure. In the present study we have not work out the degree of correlation (if any,) between the GMDS intron hypermethylation and p53 promoter hypermethylation, (as quantitative analysis of GMDS gene hypermethylation has not been studied here), although this study signifies the association between p53 promoter hypermethylation and GMDS gene hypermethylation after chronic arsenic exposure.

The intronic fragment isolated in the present study showed hypermethylation in comparison to normal unexposed subjects. This epigenetic modification may be involve in transcriptional modification and may modify the ultimate cellular level of GMDS enzyme. As it is reported that aberrant methylation of introns or intergenic regions can regulate non coding RNA function to modify the degree of transcription of a gene and the exonal expression is dependent over the local methylation status rather than the promoter region (Cheung et al. 2011). Consequences of intron methylation have also been studied by Hoivik et al. ( 2011) and Jowaed et al. ( 2010) in two separate studies where it was reported that intron methylation is associated with altered expression. Moreover, dense methylation surrounding transcription start site or near the first exon is tightly linked with gene silencing (Brenet et al. 2011).

Till to date this is the first report of GMDS intron hypermethylation in chronic arsenic exposure with and without malignancy. Reports are also unavailable regarding association between *p53*, *p16* gene hypermethylation and *GMDS* gene hypermethylation in human cancer as well as in arsenic induced cancer.

During the initial stage of the experiments we did observe some bands of hypomethylation, but we failed to clone them. It might be mentioned that in our previous investigations too, we observed far fewer hypomethylation cases. It is postulated that overexposure of arsenic and its biotransformation causes depletion of SAM, leading to hypomethylation of DNA. Hence extensive hypomethylation probably needs a very high exposure, which is achieved in artificial tissue culture systems, but rarely in real life situation. In the tissue culture experiments too, the study with cells exposed to arsenite for 2–4 weeks observed mostly hypermethylation and a few hypomethylation cases (Zhong et al. 2001). Chronic exposure of 18 weeks at low dose, on the other hand produced extensive hypomethylation and transformation in rat hepatocyte cell line (Zhao et al. 1997).

## Conclusion

To sum up, this is the first report of *GMDS* gene fragment hypermethylation in the peripheral blood leukocyte DNA of persons exposed to arsenic. To ascertain this fragment of hypermethylation as a biomarker for arsenic induced cancer and chronic arsenic exposure researchers require repetition of such work in large sample group.

## References

[CR1] Atallah R, Kalman DA (1991). Online photooxidation for the determination of organic arsenic compounds by AAS with continuous arsine generation. Talanta.

[CR2] Becker DJ, Lowe JB (2003). Fucose: biosynthesis and biological functions in mammals. Glycob.

[CR3] Bisht KK, Dudognon C, Chang WC, Sokol ES, Ramirez A, Smith S (2012). GDP-Mannose- 4,6-dehydratase is a cytosolic partner of tankyrase 1 that inhibits its poly(ADP-Ribose) polymerase activity. Mol Cell Biol.

[CR4] Brenet F, Moh M, Funk P, Feierstein E, Viale AJ, Socci ND, Scandura JM (2011). DNA methylation of the first exon is tightly linked to transcriptional silencing. PLoS ONE.

[CR5] Chanda S, Dasgupta UB, GuhaMazumder D, Gupta M, Chaudhuri U, Lahiri S, Das S, Ghosh N, Chatterjee D (2006). DNA hypermethylation of promoter of gene p53 and p16 in arsenic-exposed people with and without malignancy. Toxicol Sci.

[CR6] Cheung HH, Davis AJ, Lee TL, Pang AL, Nagrani S, Rennert OM, Chan WY (2011). Methylation of an intronic region regulates miR-199a in testicular tumor malignancy. Oncogene.

[CR7] Darnton SJ, Hardie LJ, Muc RS, Wild CP, Casson AG (2005). Tissue inhibitor of metalloproproteinase-3 (TIMP3) gene is methylated in the development of oesophageal adenocarcinoma: Loss of expression correlates with poor prognosis. Ins J Canc.

[CR8] Donohue JM, Abernathy CO, Chappel WR, Abernathy CO, Calderon RL (2001). Arsenic methylation and the S-Adenosylmethionine - mediated transmethylation/transsulfuration pathway. Arsenic Exposure and Health Effects IV.

[CR9] Eshel R, Besser M, Zanin A, Sagi-Assif O, Witz IP (2001). The FX enzyme is a functional component of lymphocyte activation. Cell Immunol.

[CR10] Fang JY, Cheng ZH, Chen YX, Lu R, Yang L, Zhu HY, Lu LG (2004). Expression of Dnmt1, demethylase, MeCP2 and methylation of tumor- related genes in human gastric cancer. World J Gastroenterol.

[CR11] Gaudet F, Hodgson JG, Eden A, Jackson-Grusby L, Dausman J, Gray JW, Leohardt H, Jaenisch R (2003). Induction of tumors in mice by genomic hypomethylation. Science.

[CR12] Goering PL, Aposhian HV, Mass MJ, Cebrian M, Beck BD, Waalkes MP (1999). The enigma of arsenic carcinogenesis: role of metabolism. Toxicol Sci.

[CR13] GuhaMazumder DN (2001). Clinical aspects of chronic arsenic toxicity. J Assoc Phys.

[CR14] GuhaMazumder DN, Haque R, Ghosh N, De BK, Santra A, Chakrabarty D, Smith AH (1998). Arsenic levels in drinking water and the prevalence of skin lesions in WestBengal, India. Int J Epidemiol.

[CR15] Haltiwanger RS (2009). Fucose is on the TRAIL of colon cancer. Gastroenterology.

[CR16] Hoivik EA, Bjanesoy TE, Mai O, Okamoto S, Minokoshi Y, Shima Y, Morohashi KI, Boehm U, Bakke M (2011). DNA methylation of intronic enhancers directs tissue-specific expression of steroidogenic factor 1/adrenal 4 binding protein (SF-1/Ad4BP). Endocrinology.

[CR17] (1997). IARC monograph on the evaluation of carcinogenic risks to humans – Overall evaluation of carcinogenicity: An update of IARC monographs 1–42. , vol 7.

[CR18] Jacobson KD, Moltanbano D (1985). The reproductive effects assessment group’s report on the mutagenicity of inorganic arsenic. Environ Mutagen.

[CR19] Jones PA, Baylin SB (2002). The fundamental role of epigenetic events in cancer. Nat Rev Genet.

[CR20] Jowaed A, Schmitt I, Kaut O, Wullner U (2010). Methylation regulates alpha-synuclein expression and is decreased in Parkinson's disease patients' brains. J Neurosci.

[CR21] Kumar S, Gadagkar SR (2001). Disparity Index: A simple statistic to measure and test the homogeneity of substitution patterns between molecular sequences. Genetics.

[CR22] Lee TC, Oshimura M, Barrett JC (1985). Comparison of arsenic-induced cell transformation, cytotoxicity, mutation and cytogenetic effects in Syrian Hamster embryo cell in culture. Carcinogenesis.

[CR23] Majumder S, Chanda S, Ganguli B, Mazumder DN, Lahiri S, Dasgupta UB (2010). Arsenic exposure induces genomic hypermethylation**.***exposure* retrieved no results. Environ Toxicol.

[CR24] Mass MJ, Wang L (1997). Arsenic alters cytocine methylation patterns of the promoter of the tumor suppressor gene p53 in human lung cells: a model for a mechanism of carcinogenecis. Mutat Res.

[CR25] Miller SS, Dykes DD, Polesky HF (1998). A simple salting out procedure for extracting DNA from human nucleated cells. Nucleic Acid Res.

[CR26] Miyoshi E, Moriwaki K, Terao N, Tan CC, Terao M, Nakagawa T, Matsumoto H, Shinzaki S, Kamada Y (2012). Fucosylation is a promising target for cancer diagnosis and therapy. Biogeosciences.

[CR27] Moriwaki K, Noda K, Furukawa Y, Ohshim K, Uchiyama A, Nakagawa T, Taniguchi N, Daigo Y, Nakamura Y, Hayashi N, Miyoshi E (2009). Deficiency of GMDS leads to escape from NK cell-mediated tumor surveillance through modulation of TRAIL signaling. Gastroenterology.

[CR28] Moriwaki K, Narisada M, Imai T, Shinzaki S, Miyoshi E (2010). The effect of epigenetic regulation of fucosylation on TRAIL-induced apoptosis. Glycoconjugate.

[CR29] Moriwaki K, Shinzaki S, Miyoshi E (2011). GMDS deficiency renders colon cancer cells resistant to TRAIL receptor and CD95 mediated apoptosis by inhibiting complex II formation. J Biol Chem.

[CR30] Nakayama K, Moriwaki K, Imai T, Shinzaki S, Kamada Y, Murata K, Miyoshi E (2013). Mutation of GDP-Mannose-4,6-Dehydratase in Colorectal Cancer Metastasis. PLoS One.

[CR31] Nandi D, Patra RC, Swarup D (2005). Effect of cysteine, methionine, ascorbic acid and thiamine on arsenic-induced oxidative stress and biochemical alterations in rats. Toxicology.

[CR32] (1999). Arsenic in Drinking water.

[CR33] Reichard JF, Puga A (2010). Effects of arsenic exposure on DNA methylation and epigenetic gene regulation. Epigenomics.

[CR34] Ren X, McHale CM, Skibola CF, Smith AH, Smith MT, Zhang L (2010). An Emerging Role for Epigenetic Dysregulation in Arsenic Toxicity and Carcinogenesis. Environ Health Perspect.

[CR35] Rossman TG, Stone D, Molina M, Troll W (1980). Absence of arsenic mutagenicity in *E.coli* and Chinese hamster cells. Environ Mutagen.

[CR36] Saha J, Gupta K, Gupta B (2013a). A new insight into the phylogeny of vascular cryptogams with special reference to *Selaginella* and *Isoetes* inferred from nuclear ITS/5.8S rDNA sequences. J Plant Biochem Biotechnol.

[CR37] Saha J, Gupta K, Gupta B (2013b). Phylogenetic analyses and evolutionary relationships of *Saraca asoca* with their allied taxa (Tribe-Detarieae) based on the chloroplast matK gene. J Plant Biochem Biotechnol.

[CR38] Sambrook J, Fritsch EF, Maniatis T vol 3, 2 edition. Edited by: Nolan C. USA: Molecular cloning: a laboratory manual cold spring harbor laboratory press; 1989.

[CR39] Slack A, Bovenzi V, Bigey P, Ivanov MA, Ramchandani S, Bhattacharya S, tenOever B, Lamrihi B, Scherman D, Szyf M (2002). Antisense MBD2 gene therapy inhibits tumorigenesis. J Gene Med.

[CR40] Sullivan FX, Kumar R, Kriz R, Stahl M, Xu GY, Rouse J, Chang XJ, Boodhoo A, Potvin B, Cumming DA (1998). Molecular Clonning of Human GDP – mannose 4, 6- Dehydratase and Recontitution of GDP-fucose Biosynthesis in Vitro. J Biol Chem.

[CR41] Tamura K, Nei M, Kumar S (2004). Prospects for inferring very large phylogenies by using the neighbor-joining method. Proc Natl Acad Sci U S A.

[CR42] Tamura K, Peterson D, Peterson N, Stecher G, Nei M, Kumar S (2011). MEGA5: Molecular Evolutionary Genetics Analysis using Maximum Likelihood, Evolutionary Distance, and Maximum Parsimony Methods. Mol Biol Evol.

[CR43] Thompson S, Cantwell BMJ, Matta KL, Turner GA (1992). Parallel changes in the blood levels of abnormally-fucosylated haptoglobin and alpha 1,3 fucosyltransferase in relationship to tumour burden: more evidence for a disturbance of fucose metabolism in cancer. Canc Lett.

[CR44] Vahter M, Chappel WR, Abernathy CO, Calderon RL (1999). Variation in human metabolism of arsenic. Arsenic Exposure and Health Effects III.

[CR45] Yuan K, Listinsky CM, Singh RK, Listinsky JJ, Siegal GP (2008). Cell surface associated alpha-L-fucose moieties modulate human breast cancer neoplastic progression. Pathol Oncol Res.

[CR46] Zhao CQ, Young MR, Diwan BA, Coogan TP, Waalkes MP (1997). Association of arsenic- induced malignant transformation with DNA hypomethylation and aberrant gene expression. Proc Natl Acad Sci.

[CR47] Zhong CX, Mass MJ (2001). Both hypomethylation and hypermethylation of DNA associated with arsenite exposure in cultures of human cells identified by methylation-sensitive arbitrarily-primed PCR. Toxicol Lett.

[CR48] Zhong CX, Wang L, Mass MJ, Abernathy CO, Calderon RL (2001). Arsenite exposure causes both hyper and hypomethylationin human cell lines in culture at low concentrations. Chappel WR.

